# MIR100HG Regulates CALD1 Gene Expression by Targeting miR-142-5p to Affect the Progression of Bladder Cancer Cells *in vitro*, as Revealed by Transcriptome Sequencing

**DOI:** 10.3389/fmolb.2021.793493

**Published:** 2022-01-21

**Authors:** Sheng Zhang, Qin Wang, Wenfeng Li, Jinzhong Chen

**Affiliations:** ^1^ Medical Oncology, Shanghai Cancer Center, Fudan University, Shanghai, China; ^2^ Shanghai University of Engineering Science, Shanghai, China; ^3^ Department of Medical Oncology, The Affiliated Hospital of Qingdao University, Qingdao, China; ^4^ State Key Laboratory of Genetic Engineering, School of Life Sciences, Fudan University, Shanghai, China

**Keywords:** MIR100HG, bladder cancer, miR-142-5p/CALD1, proliferation, migration and invasion

## Abstract

**Background/Aim:** The role of long non-coding RNA (lncRNA) and competing endogenous RNAs (ceRNA) networks in bladder cancer, especially the function of lncRNA-miRNA-mRNA regulatory network in bladder cancer, are still relatively poorly understood. This research mainly used transcriptome sequencing to screen key lncRNAs and ceRNAs, explore their pathogenic mechanism in bladder cancer, and search for potential diagnostic and therapeutic targets.

**Methods:** High-throughput transcriptome sequencing, combined with the limma package, Kaplan-Meier curve analysis, lncRNA-mRNA coexpression network, univariate Cox analysis, multivariate Cox analysis, protein-protein interaction (PPI), functional enrichment, weighed gene co-expression network analysis (WGCNA), ceRNA network and quantitative PCR (qPCR) analyses were performed to assess and screen differentially expressed lncRNAs and mRNAs. Then, the effects of MIR100HG on the proliferation, migration and invasion of the bladder cancer cell line 5,637 were evaluated using cell counting kit-8(CCK-8), wound-healing and transwell assays, respectively. A dual luciferase reporter assay was used to validate the MIR100HG/miR-142-5p and miR-142-5p/CALD1 targeting relationship, and the regulatory relationship among MIR100HG/miR-142-5p/CALD1 expression was explored using qPCR and western blot.

**Results:** A total of 127 differentially expressed lncRNAs and 620 differentially expressed mRNAs were screened. Based on the survival prognosis analysis, Cox analysis, lncRNA-mRNA network, PPI network and WGCNA, we obtained 3 key lncRNAs and 13 key mRNAs, as well as the MIR100HG/miR-142-5p/CALD1 key regulatory axis. qPCR results showed that compared with the adjacent tissues, the expression of MIR100HG and CALD1 was up-regulated, and the expression of miR-142-5p was down-regulated. Moreover, MIR100HG expression was positively correlated with the tumor grade and clinical grade of patients with bladder cancer. Overexpression of MIR100HG effectively promoted the proliferation, migration and invasion of 5,637 cells, inhibited the expression of miR-142-5p, and induced the expression of CALD1 in 5,637 cells. In addition, miR-142-5p inhibited CALD1 expression in bladder cancer cells through a direct association, and reversed the proliferation and CALD1 expression in 5,637 cells overexpressing of MIR100HG.

**Conclusion:** MIR100HG regulates CALD1 expression by targeting miR-142-5p to inhibit the proliferation, migration and invasion of bladder cancer cells. MIR100HG is an independent prognostic factor for bladder cancer, with potential as a biomarker for the diagnosis and treatment of bladder cancer.

## Introduction

Bladder cancer is a malignant tumor with high prevalence and high mortality rates. Approximately 400,000 new patients are diagnosed with bladder cancer worldwide each year, especially men, and one-third of them will die (Lenis et al., 2020; Richters et al., 2020; Teoh et al., 2020). The current treatment for bladder cancer is mainly urethral cystectomy and chemotherapy, but the therapeutic effect is effective and the prognosis is poor (Kimura et al., 2020; Tran et al., 2021). Similar to other tumors, the occurrence and development of bladder cancer are also regulated by various RNAs (Li et al., 2020a). However, the specific regulatory mechanism of these RNAs in the occurrence and development of bladder cancer is still relatively unexplained.

lncRNA is a long non-coding RNA with no protein coding function. Researchers have consistently described its very important regulatory role in various diseases (especially tumors) (Dykes and Emanueli, 2017). The regulatory mechanisms of lncRNAs in diseases mainly include chromatin modification, competing endogenous RNAs (ceRNAs), and interference with mRNA expression, *etc*. The major regulatory roles at chromatin level were dose compensation effects, genomic imprinting, chromatin modification and remodeling (Neve et al., 2021). Hu *et al* reported that lncRNA ST3Gal6-AS1 binds histone methyltransferase MLL1 and recruits it to the promoter region of ST3Gal6, inducing histone H3K4me3 modification, and then activating ST3Gal6 transcription (Hu et al., 2019). Moreover, miRNA was an important part of lncRNA function. Many lncRNA may regulate gene expression through sequencing miRNA. lncRNA could as endogenous target mimetics (eTMs), which regulate gene expression by competing with miRNAs. This mode of action was called miRNA sponge action, and the lncRNA with this function was called competitive endogenous RNA (ceRNA) (Thomson and Dinger, 2016). Liu et al. Found that lncRNA XIST competitively combined with miR-520 regulates cisplatin resistance through Bax and participates in apoptosis in p53 signaling pathway (Liu et al., 2021). Through these different mechanisms, the occurrence and progression of diseases, such as osteoarthritis, diabetes, cardiovascular diseases and cancer are initiated (Schmitz et al., 2016; Chi et al., 2019). A large number of abnormally expressed lncRNAs have been detected in bladder cancer that affect the occurrence, proliferation and migration of bladder cancer cells. For example, Zhang et al. reported that the lncRNA PCAT6 regulates the proliferation and migration of bladder cancer through the miR-143-3p/PDIA6 axis (Zhang et al., 2021). In addition, Chen et al. found that the lncRNA LNC-LBCS inhibits the self-renewal and chemotherapy resistance of bladder cancer stem cells through the epigenetic silencing of SOX2 (Chen et al., 2019). Although many differentially expressed lncRNAs have been identified in bladder cancer, relatively few studies and explanations for their functions are available (Li et al., 2020a). In addition, as mentioned above, ceRNAs are a classic regulatory mechanism of lncRNAs that regulate the expression and activity of mRNAs through targeted binding to noncoding RNAs (such as miRNAs). For example, Li et al. reported that the lncRNA LINC00461 regulates ZEB2 expression by targeting miR-30a-5p to promote the progression and malignancy of non-small cell lung cancer (Li et al., 2020b). According to Chen et al., the lncRNA MST1P2 targets the miR-133b axis to regulate the Sirt1/p53 signaling pathway and affect the chemotherapy resistance of bladder cancer to cisplatin (Chen et al., 2020a). Therefore, does this classic regulatory mechanism also exist in bladder cancer? A screen of newer key lncRNAs and analysis of their possible regulatory mechanisms is necessary to better understand the mechanisms underlying the carcinogenesis and exacerbation of bladder cancer.

In this study, we performed transcriptome sequencing of bladder cancer tissues and adjacent tissues. Then, a series of bioinformatics methods, qPCR and *in vitro* functional experiments were combined to screen and analyze key lncRNAs and their effects on bladder cancer cells. Then, dual luciferase experiments, RNA interference and qPCR were used to further study the key lncRNAs interacting with miRNAs and mRNAs, and analyze the ceRNA network. These results will provide new insights into the molecular mechanisms underlying the occurrence and development of bladder cancer, and provide new targets for the early detection and clinical treatment of patients with bladder cancer.

## Materials and Methods

### Clinical Sample Collection and Data Collection

23 bladder cancer tissues and 23 control tissues were collected during surgery (Supplementary Material S1; 3 pairs of samples were used for transcriptome sequencing and 20 pairs of samples were used for qPCR validation). Patients underwent surgical treatment at Fudan University Affiliated Cancer Hospital. Each patient provided written informed consent before the operation. The Medical Ethics Committee of Fudan University Cancer Hospital preapproved the entire research plan. The control tissue was adjacent non-cancerous bladder tissue from the same patient. After collection, all samples were washed with pre-cooled RNA-free phosphate buffer saline (PBS, Thermo, MO, United States), placed in RNAlater™ Stabilization Solution (Thermo, MO, United States), and stored at −80°C.

In addition, we downloaded the mRNA expression profiles for bladder cancer (414 bladder cancer samples and 19 control samples) and clinical features (Supplementary Material S2) from The Cancer Genome Atlas database (TCGA, https://portal.gdc.cancer.gov/).

### Analysis of Transcriptome Sequencing Data

Transcriptome sequencing was performed to identify and screen the differentially expressed lncRNAs and mRNAs in bladder cancer tissues. The MagMAX™ mirVana™ Total RNA Isolation Kit (Thermo, MO, United States) was used to extract total RNA from the sample. Subsequently, a Ribo-Zero Magnetic kit (Epicenter, United States) and magnetic stand (Thermo, MO, United States) were used to remove ribosomal RNA (rRNA) from the total RNA sample. Then, the library was constructed using the Illumina Truseq^TM^ RNA Sample Prep Kit (Illumina, MO, United States). Finally, the library was sequenced using a Novaseq 6000 SBS Kit v3-HS (200 cycles) (Illumina, United States).

After obtaining the sequencing data, we performed quality filtering of the original sequencing data to obtain high-quality clean data using the following steps: 1) remove the adapter sequence in the reads, 2) remove the bases that contain a non-AGCT at the 5′ end before cutting, 3) trim the ends of reads with lower sequencing quality (the sequencing quality value is less than Q20), 4) remove reads with a10% N content and 5) discard adapter and small fragments with a length of less than 25 bp after quality trimming are discarded. Then, the RPKM (mapped reads per thousand bases per million) value was calculated using the statistical routines provided by Hisat 2 software. After obtaining the transcript and functional annotation information in the transcriptome sequencing results, we conducted gene expression pattern classification, gene expression pattern correlation and PCA principal component analysis between samples based on the expression level of RNAs in each sample to verify the feasibility and accuracy of sequencing.

### Screening of Differentially Expressed lncRNAs and mRNAs

Firstly, the original read count was standardized (mainly to correct of sequencing depth). Then, the hypothesis test probability (*p*-value) was calculated through the statistical model. Finally, the multiple hypothesis test correction was performed using Benjiamini and Hochberg method (false discovery rate, FDR) to obtain the adjusted *p*-value (Padj value). |log2 fold change (FC) > 1 and Padj value < 0.05 were used to screening criteria of differentially expressed lncRNAs and mRNAs.

### Prognostic Analysis of Differentially Expressed lncRNAs in Patients With Bladder Cancer

GEPIA 2.0(http://gepia2.cancer-pku.cn/) was used to perform the overall survival of all differentially expressed lncRNAs in patients with bladder cancer. In addition, we download the RNA expression profiles for patients with bladder cancer and their clinical information from TCGA database and then extracted the survival data for each sample. Univariate Cox regression and multivariate Cox analyses were performed on lncRNAs related to the prognosis of patients with bladder cancer using Sangerbox 3.0(http://sangerbox.com/). Subsequently, a survival risk model was established based on the multivariate Cox analysis. The formula for calculating the prognostic risk score is risk score = *β*_1_ × expression_lncRNA1_ + *β*_2_×expression_lncRNA2_ +……+βn × expression_lncRNAn_, where *β* is the coefficient of lncRNAs obtained from multivariate Cox analysis, and expression_lncRNAn_ represents the expression level of the corresponding lncRNA. According to the median risk score, patients were divided into high-risk and low-risk groups. Then the two groups were analyzed for survival, and a receiver operating characteristic (ROC) curve was drawn.

### Analysis of lncRNA-mRNA Co-expression

Firstly, we used genome annotation and genome browser to screen mRNAs with differential expression in the upstream and downstream of lncRNAs in the range of 10 kb. Then, Pearson correlation method was used to analyze the correlation between these lncRNAs and mRNAs expression levels. The mRNAs with correlation may be the lncRNA cis regulated genes. Subsequently, for mRNAs not in the range(10 kb), Pearson correlation method was also used to analyze the correlation of their expression. These correlated mRNAs may be lncRNA trans regulated genes. All correlated mRNAs are also known as lncRNA co-expressed mRNAs. The criteria for correlation screening were Pearson’s correlation coefficient ≥0.9 and *p*-value < 0.5. The obtained lncRNAs and mRNAs were constructed in a co-expression network of lncRNAs and mRNAs visualized using Cytoscape software v3.9.0.

### Weighed Gene Co-expression Network Analysis

The mRNAs in the bladder cancer RNA expression profile in TCGA were selected using Sangerbox 3.0 (http://sangerbox.com/), and the median absolute deviation (MAD) > 50% genes were filtered by the algorithm for further analysis. Then, the goodSamplesGenes method was used to remove the outlier genes and samples. Subsequently, the Pearson’s correlation matrices were calculated with the screened sample. Then, the appropriate soft threshold power (*β*) was selected using pick Soft Threshold to establish weighted adjacency based on the scale-free topology rule matrix (WAM). Furthermore, the adjacency matrix transformed into a topological overlap measure matrix (TOM) to construct a clustering dendrogram, and the modules were identified using a dynamic tree-cut strategy. the genes closely related and co-expressed within patients with bladder cancer were classified into the same module. The execution parameter was: power = 4, minimodules = 30, deepSplit = 3, mergeCutHeight = 0.25, hub cut-off = 0.9. Finally, each module was represented by module characteristic gene (ME). The traits in this study were disease status (cancer and normal). The correlation between MEs and bladder cancer was calculated for key module selection according to the Pearson correlation coefficient. The student asymptotic *p* value was calculated using the function corPvalueStudent. the most relevant module of bladder cancer was selected for the key module (*p*-value < 0.05).

### Protein-Protein Interaction Network Construction

The PPI network was established using the STRING database v.11.5(STRING, https://string-db.org/), and the interaction pairs with confidence score ≥0.4 were retained. Then, Cytoscape software v3.9.0 was used to visualize the PPI network.

### Construction of the ceRNA Network

The key lncRNAs were picked to predict miRNAs by the means of starbase 2.0 (https://starbase.sysu.edu.cn/). Also, we explore target mRNAs of miRNAs by the starbase 2.0. Besides, Cytoscape software v3.9.0 was used to visualize the relationship of ceRNA network.

### Gene Ontology and Kyoto Encyclopedia of Genes and Genomes Analyses and Gene Set Enrichment Analysis

GO and KEGG enrichment analyses of mRNAs were performed using the database for annotation, visualization and integrated discovery v6.8 (DAVID, https://david.ncifcrf.gov/) and Metascape (https://metascape.org/). The enrichment analysis circle diagram was drawn using Sangerbox (http://sangerbox.com/). Moreover, a gene set enrichment analysis (GSEA) was performed using Sangerbox 3.0(http://sangerbox.com/).

### Real Time Quantitative PCR

The MagMAX™ mirVana™ Total RNA Isolation Kit (Thermo, MO, United States) was used to extract total RNA from the sample. Then, the RNA concentration was measured using a Nanodrop 2000 spectrophotometer. For lncRNAs and mRNAs, Prime Script RT Master Mix (Takara, China) was used for reverse transcription. The cDNA templates were then subjected to qPCR using SYBR GreenER™ qPCR Super Mix Universal (Invitrogen, MA, United States). The following primers were designed by primer 5 and synthesized by Sangon (China): MIR100HG forward primer: 5′–3′ ACT​ATG​ACA​TTC​CAG​GAA​ACC​T; MIR100HG reverse primer: 5′–3′ CCA​AGG​AGG​GAG​AAT​CCA​G; CALD1 forward primer: 5′–3′ GAA​TGC​CCA​GAA​CAG​TGT​G; CALD1 reverse primer: 5′–3′ TAT​TGT​TGG​GTC​GAA​CTC​CT; GAPDH as internal reference (GAPDH positive directional primer: 5′–3′ ATC​ATC​AGC​AAT​GCC​TCC​T; GAPDH reverse primer: 5′–3′ TTC​CAC​GAT​ACC​AAA​GTT​GTC). The 2^−ΔΔCt^ method was used calculate the relative expression level. For miRNAs, a Bulge-Loop miRNA qRT-PCR Starter Kit (RiboBio, China) was used for reverse transcription and qPCR. The miRNA primers were purchased from RiboBio (China, miR-142-5p: MQPS0000633-1-100), and U6 was used as an internal reference. The 2^−ΔΔCt^ method was used to calculate the relative expression level. Experiments were repeated 3 times.

### Cell Culture and Transfection

Human bladder cancer cells (5,637) were purchased from Beijing Beina Chuanglian Biotechnology Research Institute (China). The cells were cultured in RPMI-1640 medium (Gibco, United States) supplemented with 10% fetal bovine serum (Gibco, United States), 100 U/ml penicillin (Thermo, MA, United States) and 100 mg/ml streptomycin (Thermo, MA, United States). The culture conditions were 5% CO_2_ and 37°C.

The MIR100HG overexpression plasmid (pCDH-MIR100HG), si-MIR100HG, miR-142-5p mimic miR-142-5p inhibitor and corresponding control were synthesized by RiboBio (China) and transfected into cultured 5,637 bladder cancer cells using Lipofectamine RNAiMAX transfection reagent (Thermo, MA, United States).

### Cell Proliferation Assays

Cell proliferation was detected using the Cell Counting kit-8(CCK-8, Beyotime, China) method. After transfecting 5,637 cells with the overexpression vector or knockout vector for 24 h, the cells were collected and prepared as a suspension. Then, 5,000 cells were inoculated into each well of a 96-well plate, incubated for 48 h, and 10 μl of CCK-8 reagent were added to each well and incubated for 2 h. Finally, a multifunctional microplate reader was used to detect the OD value of each well at 450 nm and calculate the survival rate. The experiment was independently repeated 3 times.

### Wound‐healing Assay

The 5,637 cells line was inoculated into 6-well plates at a density of 5 × 10^5^ cells per well, transfected with the overexpression vector or knockout vector, and incubated for 24 h. The culture medium was removed, and the cells were scratched with a 200 μl micropipette tip. Then, the cells were rinsed with PBS (Gibco, MA, United States), and serum-free medium was added. ImageJ software was used to measure the damaged area and calculate the mobility rate = (initial wound area—48 h wound area)/initial wound area ×100%.

### Transwell Assay

Transfected 5,637 cells (5 × 10^5^ cells/well) were inoculated into the upper chamber (coated with Matrigel) (BD, NJ, United States) containing with serum-free medium, and medium containing 15% fetal bovine serum was added to the lower chamber. After 48 h of incubation, the membrane was fixed with methanol for 20 min and stained with crystal violet. The number of cells in five randomly selected fields of view was counted under an inverted microscope. The experiment was repeated three times.

### Dual Luciferase Reporter Assay

The MIR100HG wild type (WT) 3′UTR, MIR100HG mutant (MUT) 3′UTR, CALD1 wild type (WT) 3′UTR or CALD1 mutant (MUT) was inserted in the pmiR-RB-Report™ (RiboBio, China) vector and then transfected into 5,637 cells using Lipofectamine RNAiMAX reagent (Thermo, MA, United States). At the same time, the miR-142-5p mimic was transfected into the above-mentioned cells. After 48 h of incubation, dual luciferase reporter gene detection kit (Promega, WI, United States) was used to detect luciferase activity, and Renilla cell luciferase activity was used as an endogenous control.

### Western Blot

Total protein was extracted from 5,637 cells according to instructions of RIPA lysis buffer (Beyotime, China). Next, total protein concentration was determined by BCA method (Beyotime, China). Add 5×SDS loading buffer and cook for 10 min at 100°C. After cooling, it was separated by SDS-PAGE on 12% polyacrylamide gel and transferred by electrophoresis to PVDF membrane. Then, 5% skimmed milk powder (Beyotime, China) was sealed. Incubated overnight with diluted CALD1(1:10,000 diluted, Abcam, MA, United States), *β*-actin(1:10,000 diluted, Beyotime, China) primary antibody at 4°C and then incubated at room temperature for 1 h with HRP-labeled secondary antibody (Beyotime, China). Finally, DAB horseradish peroxidase chromogenic kit was used for color rendering, and the protein bands were analyzed and quantized by ImageJ software.

### Statistical Analysis

GraphPad Prism and SPSS were used for statistical analyses. Data are presented as the means ± SD. The significance of differences between two groups was tested using t tests, and the significance of differences between multiple groups was analyzed using one-way analysis of variance. *p* < 0.05 represents a significant difference.

## Result

### Summary of Transcriptome Sequencing Results

**FIGURE 1:**
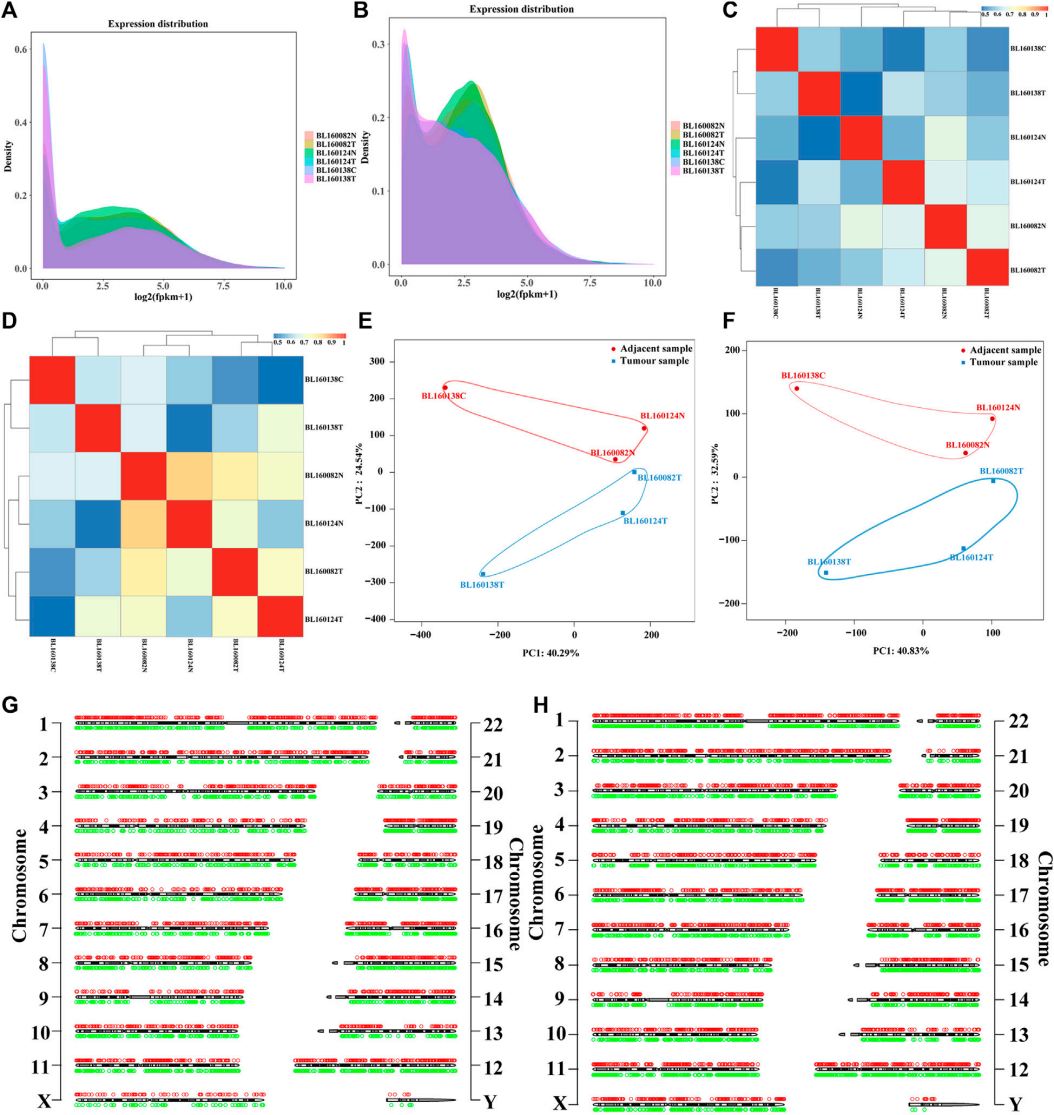
**(A)** lncRNAs expression density distribution in each sequencing sample. The peak region represents the region where the gene expression level is concentrated in the sample, which is used to compare the differences of gene expression patterns among different samples. **(B)** mRNAs expression density distribution in each sequencing sample. The peak region represents the region where the gene expression level is concentrated in the sample, which is used to compare the differences of gene expression patterns among different samples. **(C)** Correlation heat map between sequencing samples based on lncRNAs expression. **(D)** Correlation heat map between sequencing samples based on mRNAs expression. **(E)** Principal component analysis between sequencing samples based on lncRNAs expression. The blue markers were bladder cancer tissue samples, and the red markers were the corresponding adjacent tissues samples. **(F)** Principal component analysis between sequencing samples based on mRNAs expression. **(G)** The distribution of annotated lncRNAs in sequencing data on chromosomes (hg38 as reference). **(H)** The distribution of annotated mRNAs in sequencing data on chromosomes (hg38 as reference). The red circles represent up-regulated RNAs (lncRNAs and mRNAs), the green circles represent down-regulated RNAs (lncRNAs and mRNAs), and the black and white cylinder in the center represent chromosomes. The numbers on both sides were the chromosome numbers. X and Y represent chromosomes. BL160082T, BL160124T and BL160138T were bladder cancer tissue samples, while BL160082N, BL160124N and BL160138C were corresponding adjacent tissues samples.

We performed transcriptome sequencing on tissues from 3 patients with bladder cancer and 3 adjacent tissue samples to fully understand the levels of lncRNA and mRNA transcripts in the bladder. To verify the feasibility and accuracy of sequencing data, we conducted gene expression density distribution, gene expression pattern correlation and principal component analysis (PCA) principal component analysis among samples. Gene expression density distribution results showed that 3 bladder cancer tissue samples were aggregated into one group, and 3 adjacent tissue samples were also aggregated into one group. There were differences in gene expression patterns between the two groups, indicating that there was a screened differential RNAs (lncRNAs and mRNAs) in the two groups (Figures 1A,B). Gene expression pattern correlation results show that the similarity of expression patterns between samples is high, indicating that the sequencing data is reliable and the sample selection is reasonable (Figures 1C,D). PCA results showed that the samples in the same group had relatively concentrated spatial distribution, indicating that there were no outlier samples and the sequencing data could be further analyzed (Figures 1E,F). In general, we obtained 79.158 Gb of clean data (sequencing data obtained after quality control). The average clean data volume of each sample was 13.193 Gb, the Q30 base percentage was above 93.02%, and the GC content ranged from 39.35 to 46.16% (Supplementary Material S3). Then, using GRCh38 as a reference, multiple databases (NR, SwissProt and PFAM) were used to annotate all genes and transcripts. The chromosome distribution map shows the distribution and location of annotated lncRNAs and mRNAs on chromosomes (Figures 1G,H).

### Differentially Expressed lncRNAs and mRNAs in Bladder Cancer

**FIGURE 2:**
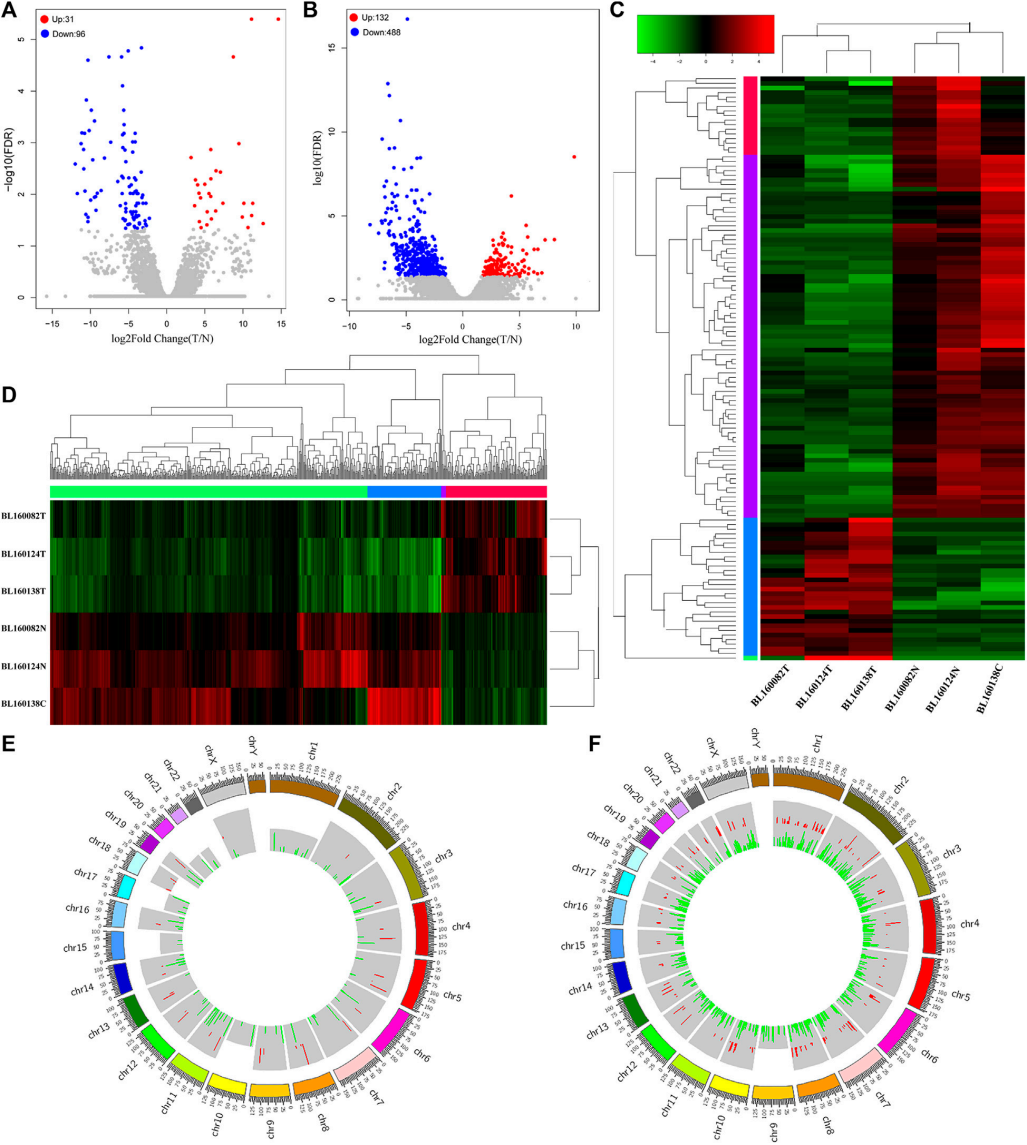
**(A)** The volcano plot showed that a total of 31 upregulated lncRNAs and 96 downregulated lncRNAs were screened out from bladder cancer sequencing data. Blue dot represented downregulated lncRNAs. Red dot represented upregulated lncRNAs. Gray dot represented normal lncRNAs. **(B)** The volcano plot showed that a total of 132 upregulated mRNAs and 488 downregulated mRNAs were screened out from bladder cancer sequencing data. Blue dot represented downregulated mRNAs. Red dot represented upregulated mRNAs. Gray dot represented normal mRNAs. **(C)** Heatmap of 127 differentially expressed lncRNAs. The diagram presents the result of a two-way hierarchical clustering of all the differentially expressed lncRNAs and samples. **(D)** Heatmap of 620 differentially expressed mRNAs. The diagram presents the result of a two-way hierarchical clustering of all the differentially expressed mRNAs and samples. **(E)** Circos histograms showed the distribution of differentially expressed lncRNAs on chromosomes (hg38 as reference). The red color of the inner circle indicates upregulated lncRNAs, and green indicates downregulated lncRNAs. The colored blocks in the outer circle represent different chromosomes, and the numbers on the outside represent the position ruler of chromosomes. **(F)** Circos histograms showed the distribution of differentially expressed mRNAs on chromosomes (hg38 as reference). The red color of the inner circle indicates upregulated mRNAs, and green indicates downregulated mRNAs. The colored blocks in the outer circle represent different chromosomes, and the numbers on the outside represent the position ruler of chromosomes. BL160082T, BL160124T and BL160138T were bladder cancer tissue samples, while BL160082N, BL160124N and BL160138C were corresponding adjacent tissues samples.

Based on the filtering criteria (Padj value < 0.05 and |log2 fold change (FC)|> 1), 127 differentially expressed lncRNAs (31 up-regulated and 96 down-regulated) and 620 differentially expressed mRNAs (132 up-regulation and 488 down-regulation) were identified. The top 10 up-regulated and down-regulated differentially expressed lncRNAs and mRNAs are shown in Tables 1, 2. The volcano plot showed the distribution of all differentially expressed lncRNAs and mRNAs (Figures 2A,B). The hierarchical clustering results (Figures 2C,D) showed that the expression patterns of all differentially expressed lncRNAs and mRNAs substantially distinguished the bladder cancer group and the adjacent control group. As shown in Figures 2E,F, the Circos diagram shows the distribution of differentially expressed lncRNAs and mRNAs on chromosomes.

**TABLE 1:** Top10 (up- and down-regulated) of differentially expressed lncRNAs in bladder cancer and adjacent tissues.

lncRNA	logFC	Gene_biotype	*p*Value	Padj
Up regulation				
LINC00967	14.446793	lincRNA	5.85E-10	4.28E-06
AC011363.1	12.456810	antisense	5.60E-04	3.90E-02
AL161630.1	11.091121	lincRNA	1.59E-04	1.58E-02
AC009652.2	10.949296	antisense	3.31E-04	2.73E-02
LINC02466	10.930985	lincRNA	1.10E-09	4.28E-06
LINC02444	10.469231	lincRNA	7.26E-04	4.65E-02
IGFL2-AS1	9.9308545	lincRNA	1.56E-04	1.58E-02
AC007128.2	9.9225318	lincRNA	1.60E-04	1.58E-02
LINC02341	9.7206037	lincRNA	3.70E-04	2.92E-02
LINC02465	9.2758257	lincRNA	3.53E-06	1.10E-03
Down regulation				
AL445465.2	−12.1814044	lincRNA	1.23E-05	0.002734055
AL136369.1	−11.9078285	antisense	8.14E-05	0.010232757
NLGN1-AS1	−11.4139638	antisense	3.43E-06	0.001101752
AC116456.1	−11.3044163	antisense	1.40E-06	0.000681887
AP003548.1	−11.0832922	lincRNA	4.96E-06	0.001434254
AL355073.2	−11.0583421	antisense	1.57E-05	0.003396762
NCAM1-AS1	−10.9532117	antisense	1.65E-06	0.000697001
AC010082.1	−10.9006501	antisense	6.32E-05	0.009137361
AP002833.1	−10.7851825	lincRNA	3.09E-04	0.025968474
PGR-AS1	−10.7221019	processed transcript	2.02E-07	0.000157829

**TABLE 2:** Top10 (up- and down-regulated) of differentially expressed mRNAs in bladder cancer and adjacent tissues.

mRNA	logFC	Gene_biotype	*p*Value	Padj
Up regulation				
UGT1A10	9.700965978	protein coding gene	1.92E-12	3.65E-09
EN1	7.950228894	protein coding gene	1.65E-06	0.000309377
SULT1E1	7.126220052	protein coding gene	1.75E-06	0.000320277
AC137834.1	6.841638845	protein coding gene	9.98E-04	0.02982833
LHX5	6.49225441	protein coding gene	9.98E-06	0.001169272
NXPH1	6.455902707	protein coding gene	1.17E-03	0.033339275
RPS10-NUDT3	6.158335711	protein coding gene	1.05E-05	0.001180991
PCDH20	6.135487131	protein coding gene	8.47E-04	0.026327946
AC013470.2	5.69963623	protein coding gene	4.13E-04	0.016173879
SCEL	5.655882454	protein coding gene	1.20E-03	0.033594625
Down regulation				
SCN11A	−8.30830531	protein coding gene	1.34E-07	4.00E-05
C2orf40	−7.55122607	protein coding gene	5.41E-07	0.000128779
CDH19	−7.29773672	protein coding gene	6.80E-08	2.41E-05
ATP1A2	−7.23934239	protein coding gene	1.03E-13	3.14E-10
VIT	−7.23112716	protein coding gene	9.65E-08	3.19E-05
RBP4	−7.05699725	protein coding gene	4.25E-04	0.016396553
PCSK2	−7.05228978	protein coding gene	2.17E-10	2.07E-07
XPNPEP2	−7.00307723	protein coding gene	1.32E-04	0.007082633
LRRC3B	−6.99022528	protein coding gene	1.56E-09	9.13E-07
SCARA5	−6.97780004	protein coding gene	6.02E-08	2.18E-05

### Prognostic Analysis of lncRNAs in Patients With Bladder Cancer

**FIGURE 3:**
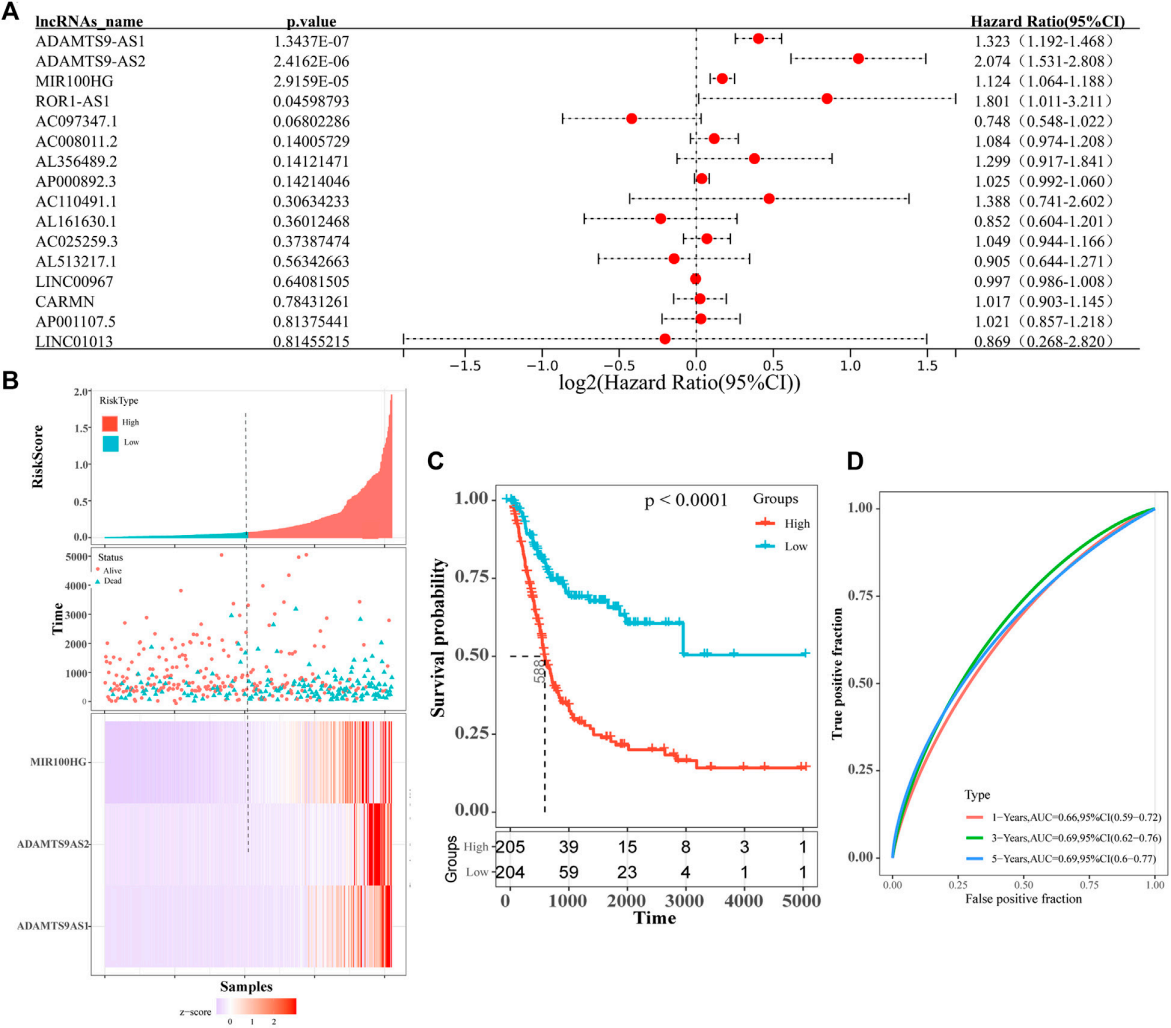
**(A)** Forest map shows univariate COX analysis results of lncRNAs associated with prognosis of bladder cancer patients. The red dots represent the Hazard ratio of univariate Cox regression analysis, and the two ends of the red dot represent the maximum and minimum values of the 95% confidence interval(95%Cl). **(B)** The distribution of risk scores (picture above), patient relapse status (picture middle) and gene expression levels (picture above) of 3-lncRNA signature (ADAMTS9-AS1, ADAMTS9-AS2 and MIR100HG). The black dotted line represents the median risk score cut-off dividing patients into low- and high-risk groups. **(C)** Kaplan–Meier plot of overall survival for patients in low-risk and high-risk groups. The black dotted line represents median survival time. The table at the bottom is the risk table, which counts the sample size for each risk group. Significant prognostic differences(*p*-value) between the two groups were assessed by logrank test(*p*-value<0.0001). **(D)** ROC curve for the 1 year (red curve), 3 years (green curve) and 5 years (blue curve) survival prediction by the 3-lncRNA signature (ADAMTS9-AS1, ADAMTS9-AS2 and MIR100HG).

All differentially expressed lncRNAs were analyzed for their correlations with survival, and 16 lncRNAs that were significantly related to the overall survival of patients with bladder cancer were identified (logrank test *p*-value <0.05). Among them, AL513217.1, AC097347.1, AL161630.1 and LINC00967 were associated with longer overall survival of patients with bladder cancer (hazard ratio <1). Other factors were related to shorter overall survival of patients with bladder cancer (hazard ratio >1, Supplementary Table S1). Subsequently, we performed a univariate Cox analysis on the 16 prognostic-related lncRNAs that were screened, and obtained 4 more significant prognosis lncRNAs (ADAMTS9-AS1, ADAMTS9-AS2, MIR100HG and ROR1AS1) (Figure 3A; Table 3). Then, we continued to perform a multivariate Cox analysis and identified 3 lncRNAs (ADAMTS9-AS1, ADAMTS9-AS2 and MIR100HG) that were more relevant to the prognosis of patients with bladder cancer (Table 3). Furthermore, based on the multifactor Cox analysis and the expression profiles of 3 lncRNAs, we constructed a prognostic risk model. The risk scoring formula was 0.1815×expression_ADAMTS9-AS1_+ 0.5605×expression_ADAMTS9-AS2_+ 0.0670 ×expression_MIR100HG_. According to the median risk score, the patients were divided into high-risk groups and low-risk groups. As shown in Figure 3B, the patients who died were mainly assigned to the high-risk areas group. At the same time, the expression heatmap of ADAMTS9-AS1, ADAMTS9-AS2 and MIR100HG also showed high expression in the high-risk areas. This finding is consistent with the results of the survival analysis. In addition, the results of the prognostic analysis of the high-risk and low-risk groups showed that the high-risk group had a worse prognosis (*p*-value<0.05, Figure 3C). We conducted a ROC curve analysis to verify the clinical value and stability of the risk model. The areas under the ROC curve for the 1 year, 3 years, and 5 years prognostic models were 0.66, 0.69, and 0.69, respectively (Figure 3D). Based on these data, the 3 lncRNAs selected (ADAMTS9-AS1, ADAMTS9-AS2 and MIR100HG) may be independent prognostic factors for patients with bladder cancer and have the potential to be used as prognostic markers.

**TABLE 3:** Univariate (A) and multivariate(B) Cox analyses of overall survival in bladder cancer.

ID	lncRNAs	HR (95%CI)	*p*-value
A			
ENSG00000257042	AC008011.2	1.084 (0.974–1.208)	0.140057286
ENSG00000260947	AL356489.2	1.299 (0.917–1.841)	0.141214714
ENSG00000254510	AP001107.5	1.021 (0.857–1.218)	0.813754407
ENSG00000241158	ADAMTS9-AS1	1.323 (1.192–1.468)	1.34374E-07
ENSG00000261292	AC110491.1	1.388 (0.741–2.602)	0.306342327
ENSG00000228495	LINC01013	0.869 (0.268–2.820)	0.814552153
ENSG00000249669	CARMN	1.017 (0.903–1.145)	0.784312615
ENSG00000241684	ADAMTS9-AS2	2.074 (1.531–2.808)	2.41618E-06
ENSG00000259884	AC025259.3	1.049 (0.944–1.166)	0.373874739
ENSG00000223774	AL513217.1	0.905 (0.644–1.271)	0.563426632
ENSG00000223949	ROR1-AS1	1.801 (1.011–3.211)	0.045987932
ENSG00000255248	MIR100HG	1.124 (1.064–1.188)	2.91587E-05
ENSG00000280143	AP000892.3	1.025 (0.992–1.060)	0.142140463
ENSG00000231217	AC097347.1	0.748 (0.548–1.022)	0.068022855
ENSG00000233569	AL161630.1	0.852 (0.604–1.201)	0.360124685
ENSG00000253138	LINC00967	0.997 (0.986–1.008)	0.640815048
**B**			
ENSG00000241158	ADAMTS9AS1	1.748 (1.217–2.510)	0.002505851
ENSG00000241684	ADAMTS9AS2	1.184 (1.035–1.355)	0.013877292
ENSG00000255248	MIR100HG	1.068 (1.003–1.138)	0.039284209
ENSG00000223949	ROR1AS1	1.338 (0.742–2.413)	0.33337269

### Co-Expression Relationships Between Key lncRNAs and Differentially Expressed mRNAs

**FIGURE 4:**
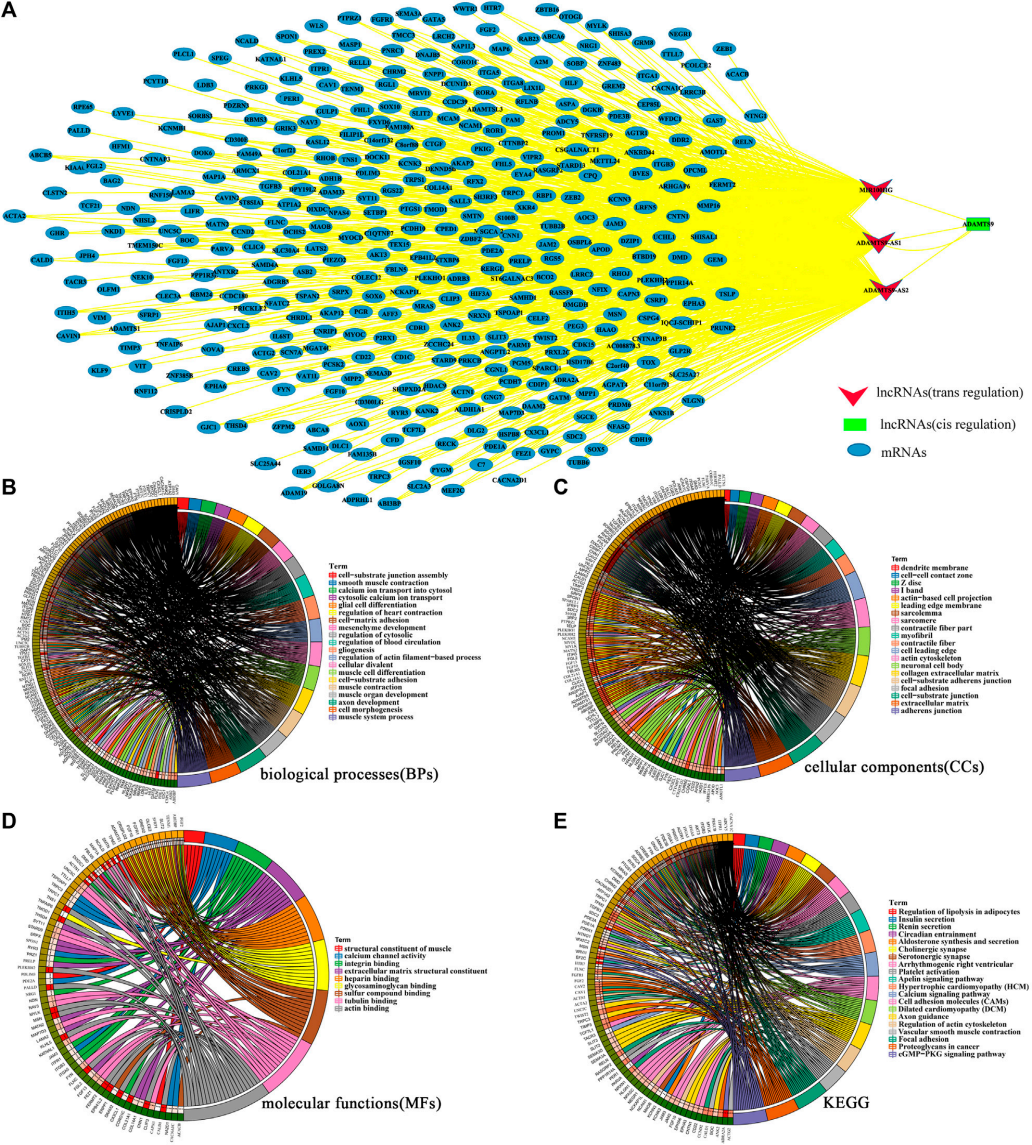
**(A)**. The lncRNA-mRNA coexpression network of key lncRNAs and key mRNAs. The red invaginated triangle represents *trans*-regulated lncRNAs. The green squares represent cis regulated lncRNAs. The blue oval represents mRNAs. (There were two different regulation modes for the co expression of lncRNA and mRNA. One was cis regulation. In this regulation mode, lncRNA only acts with mRNAs within 10 kb upstream and downstream, and their expression was correlated. The other was trans regulation, which was aimed at mRNAs that are not within the distance range but have correlation in expression.) **(B,C,D,E)** Functional enrichment analysis of mRNAs in the lncRNA-mRNA coexpression network. The possible biological processes **(B)**, cell components **(C)**, molecular functions **(D)** and signal pathways **(E)** were analyzed. The circle diagram consists of two parts, the left half represents the mRNAs analyzed, and the right half represents the biological functions enriched by mRNAs. *p*-value of <0.05 and False Discovery Rates (FDR) < 0.25 were considered statistically significant.

We analyzed the potential regulatory network between lncRNAs and differentially expressed mRNAs to further understand the biological functions of the 3 key lncRNAs (ADAMTS9-AS1, ADAMTS9-AS2 and MIR100HG) related to prognosis. As shown in Supplementary Material S4, we obtained three key lncRNAs cis regulated and trans regulated mRNAs.Then, Cytoscape was used to construct and visualize the lncRNA-mRNA co-expression network. As shown in Figure 4A, the lncRNA-mRNA co-expression network consisted of 391 nodes and 900 edges, including 388 trans target mRNAs (blue oval) and 1 cis target (green square). Subsequently, we analyzed the enriched functions and pathways of the 389 target mRNAs. The results showed that the main enriched biological functions (BPs) were muscle contraction and development, cell matrix adhesion and blood circulation regulation (Figure 4B). The main enriched cell components (CCs) were processes of cell matrix connection, nerve formation and contraction (Figure 4C). The main enriched molecular functions (MFs) were protein binding and ion channel activity (Figure 4D). In addition, the main enriched KEGG were the cGMP-PKG signaling pathway, vascular smooth muscle contraction, focal adhesion, proteoglycans in cancer and cell adhesion molecules (CAMs) (Figure 4E).

### Identification of Key mRNA Modules Using WGCNA

**FIGURE 5:**
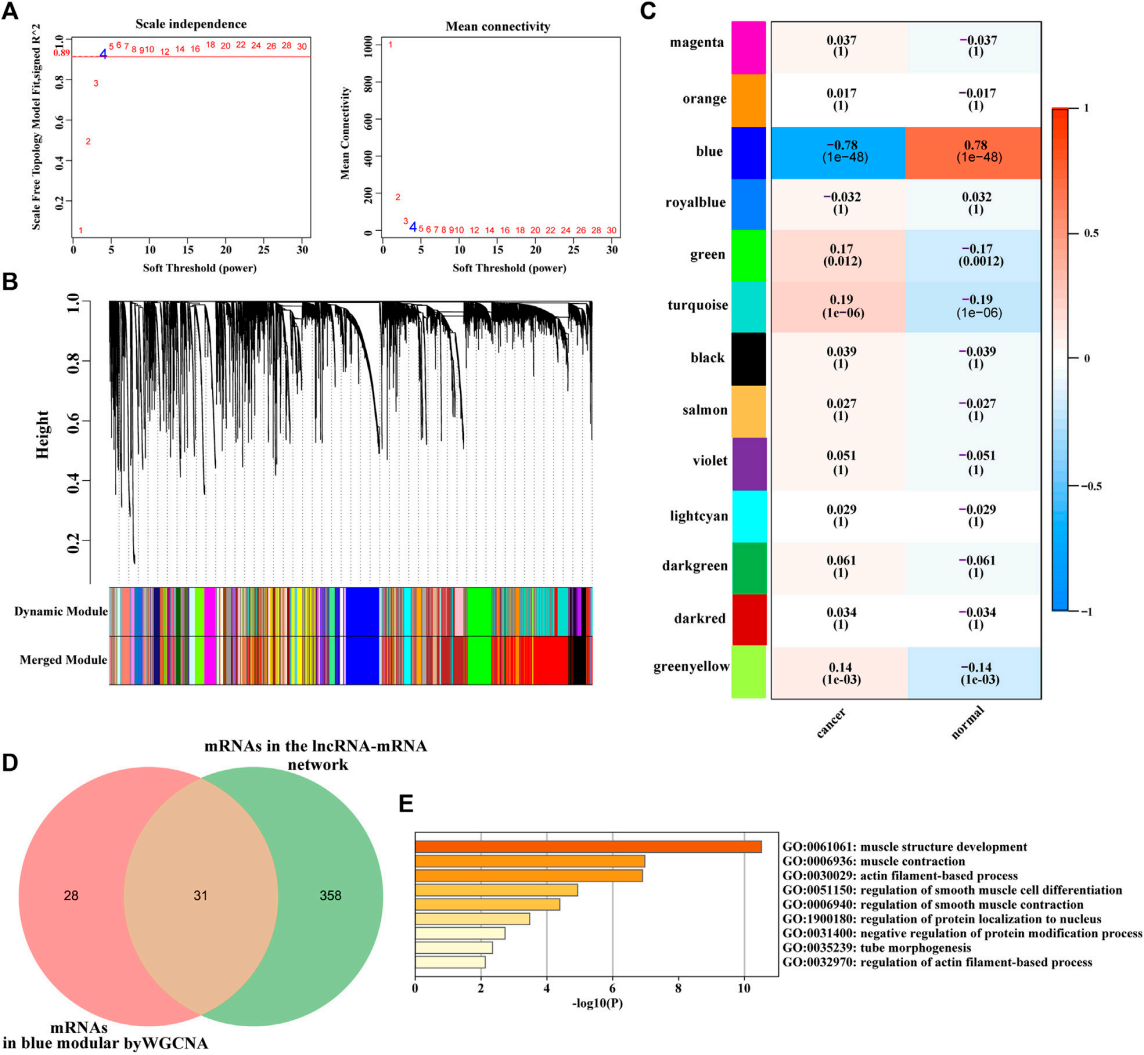
**(A)** (left): Analysis of the scale-free fit index for various soft-thresholding powers. **(A)**(right): Analysis of the mean connectivity for various soft-thresholding powers. The red line represented the square of the correlation coefficient, 0.89, and the first point above red line was the soft threshold β = 4. **(B)** Hierarchical cluster analysis of mRNAs (the median absolute deviation (MAD)>50%) was conducted to detect co-expression clusters with corresponding color assignments. Each color represents a module in the constructed gene co-expression network by WGCNA. **(C)** Heat map of the correlation between modules and traits. The box includes the Pearson correlation coefficient (PCC) and the corresponding *p*-value. The box color (ranges from blue to red) is correlated with the PCC value (range from –1 to 1). The red box indicates the module is positively correlated with traits, while the blue box indicates the module is negatively correlated with traits. The traits in this study were bladder cancer status (cancer and normal). **(D)** Venn diagram of differentially expressed mRNAs in lncRNA-mRNA coexpression network and mRNAs screened via WGCNA in TCGA bladder cancer dataset. **(E)** The Bar graph shows the enriched terms of 31 mRNAs using Metascape(http://metascape.org/), colored by *p*-values. Adj.*p*-value <0.05 were considered statistically significant. The darker the color, the greater the *p*-value.

After removing abnormal samples and screening genes, 433 samples with 11,348 genes were extracted from the TCGA bladder cancer dataset to construct a weighted gene co-expression network. The analysis results show that the co-expression network conformed to unsigned network. Soft threshold was selected based on the square of correlation coefficient 0.89 (*β* = 4, Figure 5A). Based on the obtained soft threshold (*β* = 4), 35 different coexpression modules were completely identified by dynamic tree cutting (Figure 5B). In order to further understand the correlation between modules and bladder cancer, we obtained the correlation between module eigengene and tumor status. As shown in Figure 5C, the blue module (correlation: 0.78, *p*-value: 1e−48) were the most significant modules among the modules associated with bladder cancer, which contained 59 mRNAs. Therefore, the blue module is considered a key module for bladder cancer. Subsequently, mRNAs in the blue module and mRNAs in the lncRNA-mRNA network were comparative analysis using Venn diagram, and 31 common mRNAs were obtained (Figure 5D). The GO and KEGG enrichment analyses showed that the 31 common mRNAs were mainly involved in muscle contraction, protein binding, cell matrix connection, vascular smooth muscle contraction pathway and calcium signaling pathway (Figure 5E).

### Construction of the Protein-Protein Interaction Network of 31 Common mRNAs

**FIGURE 6:**
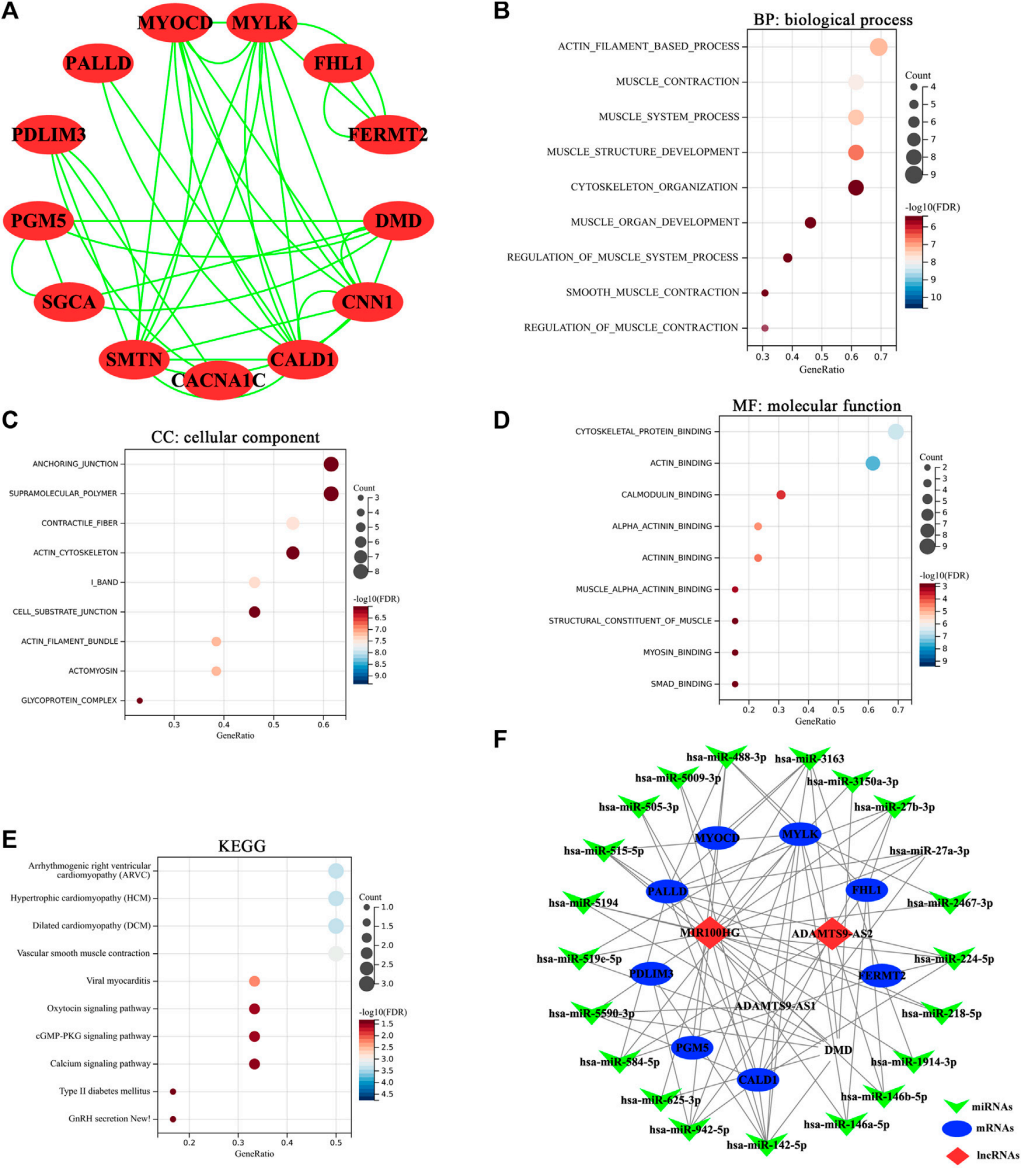
**(A)** Diagram of protein-protein interaction (PPI) network. Each dot represented a node. The more line segments connected to the node, the greater the degree of this node. **(B)** The top 10 significantly enriched biological process terms of mRNAs in PPI network. **(C)** The top 10 significantly enriched cellular component terms of mRNAs in PPI network. **(D)** The top 10 significantly enriched molecular function terms of mRNAs in PPI network. **(E)** The 10 significantly enriched KEGG pathways of mRNAs in PPI network. Horizontal axis: the number of enriched genes; vertical axis: the corresponding biological process; Adj.*p*-value <0.05 were considered statistically significant. The color of the bubble indicates the adjusted *p*-value of the GO terms or pathways, and the size of the bubble signifies the number of genes associated with a term. **(F)** The lncRNA-miRNA-mRNA competing endogenous RNA (ceRNA) network in bladder cancer. The invaginated triangles indicate miRNAs in green, ellipses represent mRNAs in blue and rhombus represent lncRNAs in red.

The 31 common genes were selected to construct PPI network. Interaction pairs with confidence score ≥0.4 were visualized using Cytoscape software v 3.9.0 (Figure 6A), which consisted of 13 nodes and 38 edges. As showed in Figure 6A, there were 13 interacting genes among the 31 common genes. Among them, CALD1,CNN1, MYLK and SMTN have the highest node degree, all of which were 10. Subsequently, we performed functional enrichment analysis on 13 interacting genes. The enrichment results showed that these 13 genes were mainly involved in muscle contraction, muscle cell differentiation, ion channel regulation and angiogenesis (Figures 6B,C,D). The main signaling pathways involved were vasoconstriction, MAPK signaling pathway and cancer-related proteoglycan (Figure 6E). The results suggested that these 13 genes, including CACNA1C, PALLD, FHL1, PDLIM3, SGCA, PGM5, FERMT2, DMD, MYOCD, CALD1, CNN1, MYLK and SMTN, were more crucial to the pathogenesis of bladder cancer.

### Construction of the ceRNA Network of Key RNAs (lncRNAs and mRNAs)

By online prediction of target genes, we obtained 21 common miRNAs (the target miRNAs of 3 key lncRNAs and the miRNAs targeting the 13 key mRNAs) (Table 4). Subsequently, we built the ceRNA network on the basis of the 3 key lncRNAs, 21 miRNAs and13 key mRNAs. As shown in Figure 6F, 3 lncRNAs and 21 miRNAs were paired into 23 lncRNAs–miRNAs interactions, whereas 21 miRNAs and 9 mRNAs were matched to form 55 pairs of miRNAs–mRNAs interactions. According to the degree of the node, MIR100HG had the highest node degree of 19 in lncRNAs category. miR-142-5p had the highest node degree of 7 in miRNAs category. CALD1 had the highest node degree (degree = 9) and topological coefficient (topological coefficient = 0.358) in mRNAs category (Supplementary Material S5). Therefore, the MIR100HG/miR-142-5p/CALD1 axis was chosen for further analysis.

**TABLE 4:** RNAS (lncRNAs, miRNAs and mRNAs) in ceRNA network.

lncRNA	miRNA (targets of lncRNA)	mRNA (targets of miRNA)
MIR100HG	hsa-miR-27a-3p	CALD1, PALLD, FHL1
	hsa-miR-224-5p	CALD1, PALLD, FERMT2
	hsa-miR-27b-3p	CALD1, PALLD, FHL1
	hsa-miR-142-5p	CALD1, MYLK, PDLIM3, PALLD, DMD
	hsa-miR-505-3p	CALD1
	hsa-miR-942-5p	CALD1, MYLK, PDLIM3, FHL1
	hsa-miR-3163	CALD1, MYOCD, PALLD, DMD, FERMT2
	hsa-miR-5590-3p	CALD1, DMD
	hsa-miR-146a-5p	MYLK, FHL1
	hsa-miR-146b-5p	MYLK, FHL1
	hsa-miR-515-5p	MYLK, PALLD, DMD
	hsa-miR-519e-5p	MYLK, DMD
	hsa-miR-488-3p	MYLK, PALLD, PGM5, FHL1
	hsa-miR-2467-3p	MYLK
	hsa-miR-218-5p	PALLD
	hsa-miR-5194	DMD, FERMT2
	hsa-miR-3150a-3p	DMD
	hsa-miR-1914-3p	FERMT2
	hsa-miR-625-3p	DMD
ADAMTS9-AS1	hsa-miR-5590-3p	CALD1, DMD
	hsa-miR-142-5p	CALD1, MYLK, PDLIM3, PALLD, DMD
	hsa-miR-5009-3p	CALD1
ADAMTS9-AS2	hsa-miR-584-5p	MYLK, PALLD, FERMT2

Note: Results predicted by starBase 2.0 (http://starbase.sysu.edu.cn/) online.

### qPCR Verification and Correlation Analysis Between MIR100HG Expression and Clinicopathological Parameters of Bladder Cancer

**FIGURE 7:**
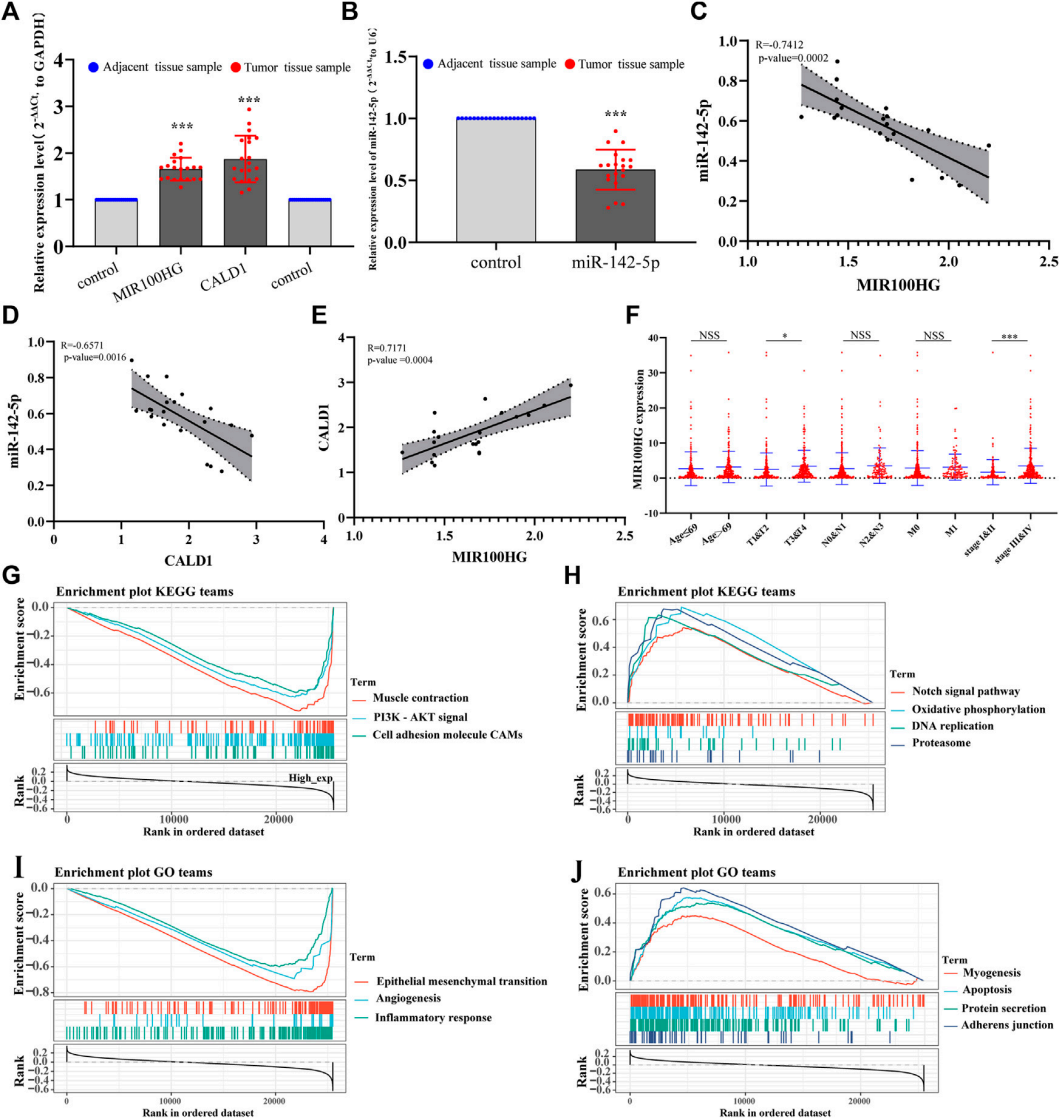
**(A)** The relative expression levels of MIR100HG and CALD1 in bladder cancer tissues and adjacent tissues using qPCR. The blue dots represent adjacent tissue samples of bladder cancer. The red dots represent bladder cancer tissue samples. The expression level of GAPDH was used as an internal reference. The data are shown as the mean ± standard deviation. The left “control” on the *X* axis represents the relative expression of MIR100HG in the adjacent tissue samples (as a reference, the expression value was 1). The right “control” represents CALD1 relative expression in the adjacent tissue samples (as a reference, the relative expression value was 1). **(B)** The expression levels of miR-142-5p in bladder cancer tissues and adjacent tissues using qPCR. The blue dots represent adjacent tissue samples of bladder cancer. The red dots represent bladder cancer tissue samples. The expression level of U6 was used as an internal reference. The data are shown as the mean ± standard deviation. The “control” on the *X* axis represents the relative expression of miR-142-5p in the adjacent tissue samples (as a reference, the relative expression value was 1). **(C,D,E)** Correlation analysis of MIR100HG, miR-142-5p and CALD1 relative expression levels in 20 pair bladder cancer tissue samples and adjacent tissues samples. **(F)** Correlations between MIR100HG expression and clinicopathological characteristics of bladder cancer. The red dots represent MIR100HG expression in TCGA- BLCA samples. “Age ≤69 and age >69” represents the age group of TCGA-BLCA, and 69 is the median age value of patients. “T, N and M” represents the tumor pathological stage of patients in TCGA-BLCA, “&” represents the integration of two data into one data. “Stage” represents the tumor tissue stage of patients in TCGA-BLCA. **(G,H,I,J)** Gene set enrichment analysis (GSEA) of MIR100HG in TCGA-BLCA patients. **(G)**: KEGG terms with negative correlation with MIR100HG expression. **(H)**: KEGG terms with positive correlation with MIR100HG expression. **(I)**: GO terms with negative correlation with MIR100HG expression. **(J)**: GO terms with positive correlation with MIR100HG expression. ***p-value < 0.001. * *p*-value < 0.05.NSS: no statistical significance.

As a method to verify the key RNAs identified using transcriptome sequencing, we collected 20 pairs of clinical samples (20 bladder cancer tissue samples and 20 corresponding tumor-adjacent tissues, Supplementary Material S1), and performed qRT–PCR to detect the expression levels of MIR100HG, miR-142-5p and CALD1 in clinical tissues. The trends in the expression of MIR100HG and CALD1 were consistent with the transcriptome sequencing results, and their levels were up-regulated in bladder cancer (Figure 7A). The trend of miR-142-5p expression was consistent with the results of biosynthesis results, and its expression was down-regulated in bladder cancer (Figure 7B). A correlation analysis of the expression levels of MIR100HG, miR-142-5p and CALD1 showed that miR-142-5p was negatively correlated with CALD1 and MIR100HG expression levels, and the expression levels of MIR100HG and CALD1 were positively correlated (Figures 7C,D,E).

In addition, after downloading the clinical data for patients with bladder cancer from TCGA, we evaluated the relationship between MIR100HG expression and various clinicopathological parameters patients with bladder cancer. The increase in MIR100HG expression was significantly correlated with the tumor histological grade (pathological-T stage, *p*-value <0.05) and clinical stage (*p* < 0.05, Figure 7F). Compared with patients with bladder cancer presenting low MIR100HG expression levels, patients with bladder cancer presenting high MIR100HG expression levels were more likely to have a more advanced tumor status, grade and stage, which leads to disease progression and poor prognosis. This result is consistent with the prognostic analysis of MIR100HG. Moreover, gene set enrichment analysis (GSEA) further showed that MIR100HG may be involved in the epithelial-mesenchymal transition, angiogenesis, apoptosis, muscle cell contraction, PI3K-AKT signaling pathway, cell adsorption, Notch signaling pathway, and amino acid metabolism. These biological processes are related to the occurrence, invasion and metastasis of bladder cancer (Figures 7G,H,I,J). Therefore, we must further verify the effect of MIR100HG on the progression of bladder cancer by performing additional experiments.

### The Effect of MIR100HG on the Proliferation, Migration and Invasion of Bladder Cancer Cells (5,637 Cells)

**FIGURE 8:**
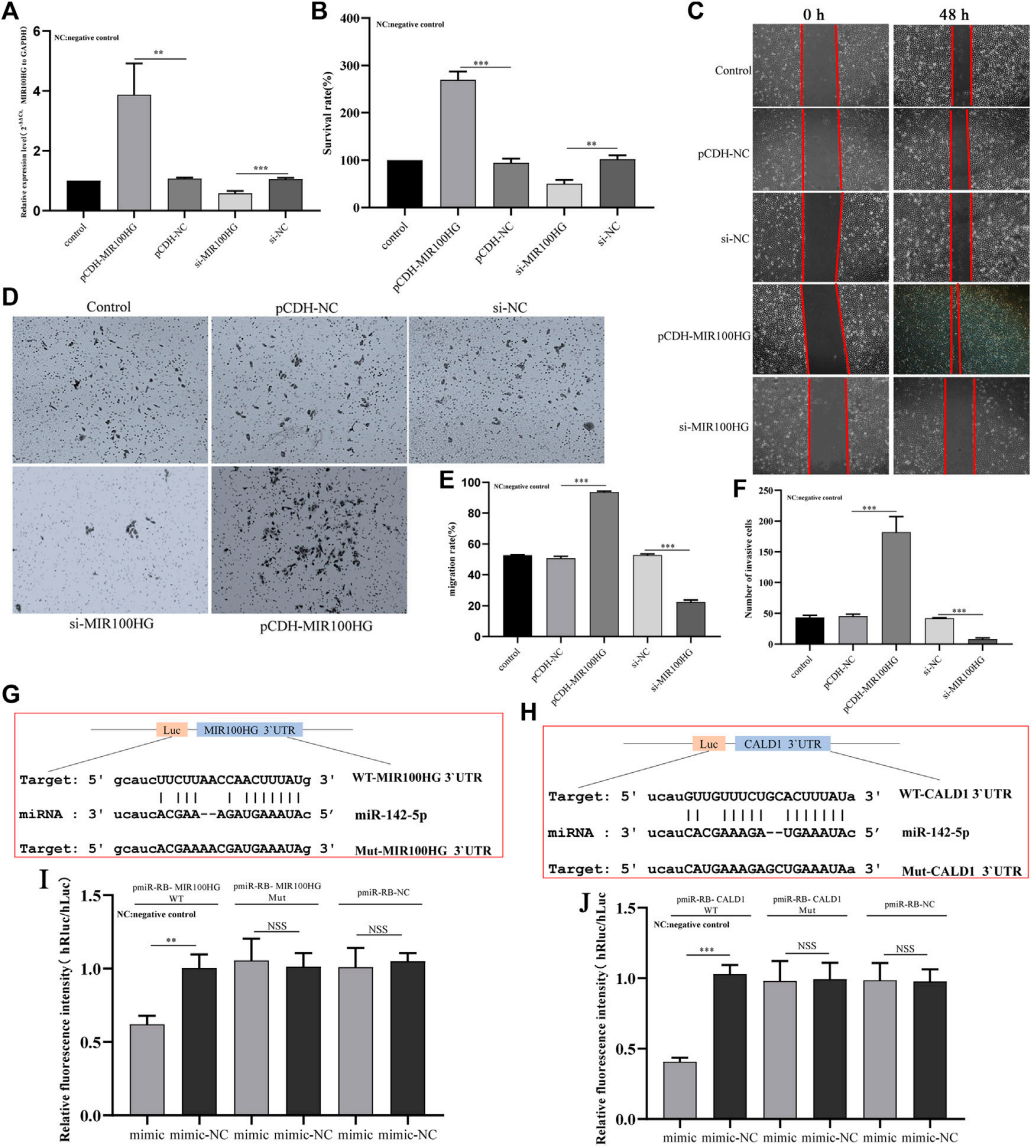
**(A)** Analysis of the effects of MIR100HG knockdown or overexpression on 5,637 cells using qPCR. The expression level of GAPDH was used as an internal reference. **(B)** Analysis of the effects of MIR100HG knockdown or overexpression on the proliferation of 5,637 cells using the CCK-8 assay. **(C,E)** Analysis of the effects of MIR100HG knockdown or overexpression on the migration of 5,637 cells using the wound healing assay. **(D,F)** Analysis of the effects of MIR100HG knockdown or overexpression on the invasion of 5,637 cells using transwell assays. **(G)** Sequence of miR-142-5p and MIR100HG 3′UTR binding sites. **(H)** Sequence of miR-142-5p and CALD1 3′UTR binding sites. **(I)** Results of the dual luciferase assay for miR-142-5p and MIR100HG. **(J)** Results of the dual luciferase assay for miR-142-5p and CALD1. NC stands for negative control. pCDH-NC represents negative plasmid of pCDH- vector, and has no effect on cell biological function after transfection. Si-NC represents the negative control of si-RNA, which has no effect on cell biological function after transfection. “Control” refers to the cell group without any plasmid transfection, which is only used as a reference for the analysis of the experimental group. The data are shown as the mean ± standard deviation. WT: wild type. Mut: mutant type. Luc: Luciferase. “***” *p*-value <0.001, “**” *p*-value <0.01, “NSS”: no statistical significance.

We synthesized the MIR100HG overexpression vector pCDH-MIR100HG and the knockout vector si-MIR100HG to further study the biological functions of MIR100HG in bladder cancer. First, the transfection efficiency of the two vectors in 5,637 cells was tested using qPCR. MIR100HG modulation was observed in 5,637 cells (Figure 8A). CCK-8 results showed that MIR100HG overexpression significantly increased the proliferation of 5,637 cells, and MIR100HG silencing exerted opposite effect on proliferation (Figure 8B). Similarly, wound-healing experiments and Transwell experiments showed that MIR100HG overexpression promoted the migration and invasion of 5,637 cells, where MIR100HG knockout significantly inhibited migration and invasion (Figures 8C,D,E,F). Based on these results, high MIR100HG expression promotes the progression of bladder cancer cells *in vitro*, consistent with the results from the correlation analysis of clinicopathological parameters.

### MIR100HG Regulates CALD1 Expression in Bladder Cancer Cell Processes by Targeting miR-142-5p

**FIGURE 9:**
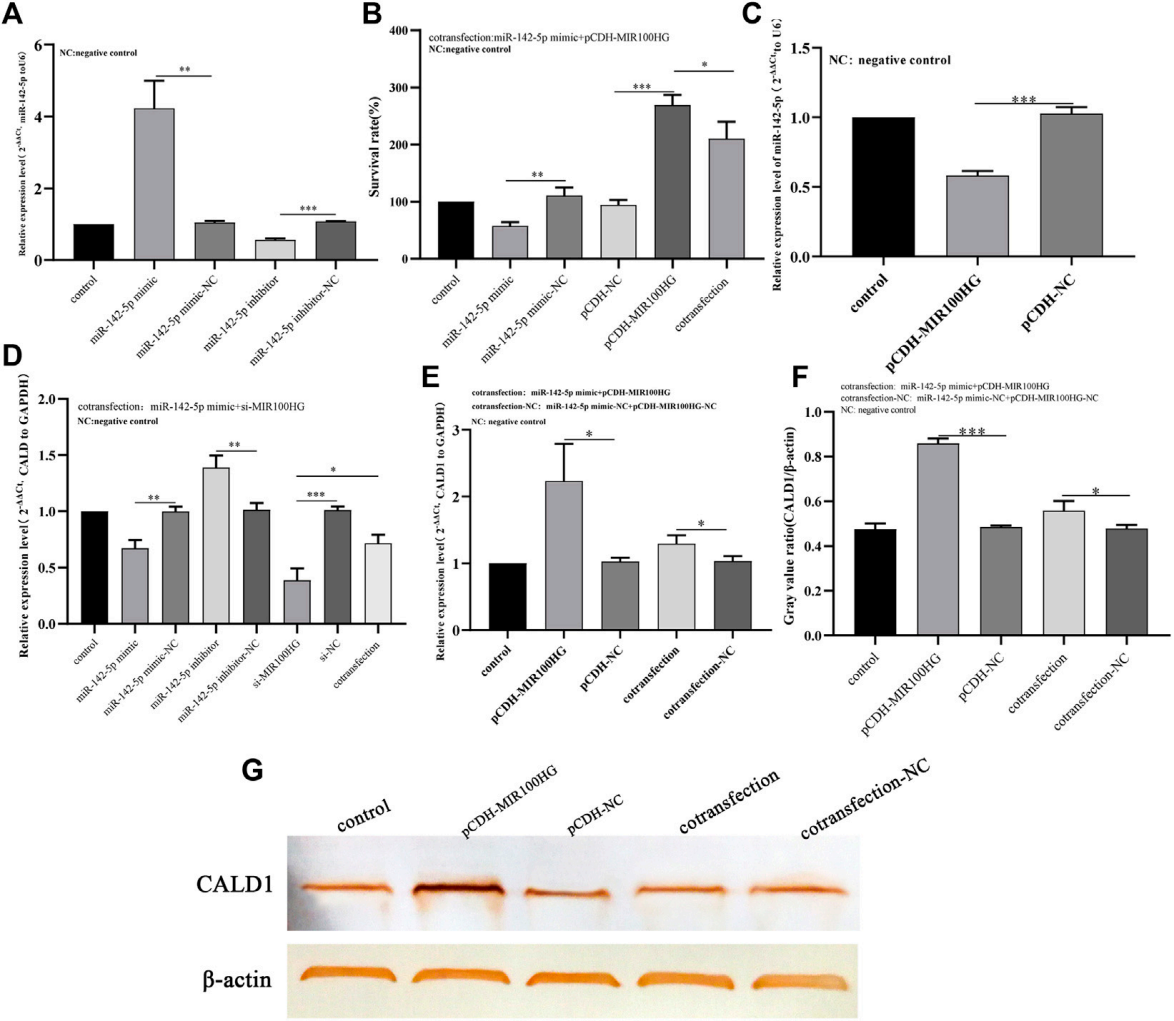
**(A)** Analysis of the effects of miR-142-5p knockdown or overexpression in 5,637 cells using qPCR. The expression level of U6 was used as an internal reference. **(B)** The effect of miR-142-5p interference with MIR100HG on the proliferation of 5,637 cells using CCK8 assay. **(C)** Analysis of effect of MIR100HG overexpression on endogenous miR-142-5p expression in 5,637 cells using qPCR. The expression level of U6 was used as an internal reference. **(D)** Analysis of effect of miR-142-5p interference with MIR100HG on CALD1 expression in 5,637 cells using qPCR. The expression level of GAPDH was used as an internal reference. **(E)** Analysis of effect of miR-142-5p targeted by MIR100HG on CALD1 expression level in 5,637 cells using qPCR. The expression level of GAPDH was used as an internal reference. **(F,G)** Analysis of effect of miR-142-5p targeted by MIR100HG on CALD1 protein level in 5,637 cells using western blot. The protein level of *β*-actin was used as an internal reference. NC stands for negative control. pCDH-NC represents negative plasmid of pCDH- vector, and has no effect on cell biological function after transfection. Si-NC represents the negative control of si-RNA, which has no effect on cell biological function after transfection. “Control” refers to the cell group without any plasmid transfection, which is only used as a reference for the analysis of the experimental group. The data are shown as the mean ± standard deviation. Cotransfection: The two target plasmids were transferred into cells at the same time. Cotransfection-NC: The negative control of the two target plasmids were transferred into the cells at the same time. “***” *p*-value <0.001,”**” *p*-value <0.01,”*” *p*-value <0.05.

We first performed a dual luciferase experiment to further understand the regulatory relationship of the MIR100HG/miR-142-5p/CALD1 axis, The online platform starBase predicted the binding sites in the MIR100HG 3′UTR for miR-142-5p, and the binding sites for miR-142-5p in the CALD1 3′UTR. The binding site sequences are shown in Figures 8G,H. The miR-142-5p mimic induced a significant decrease in luciferase activity in cells transfected with WT-MIR100HG and WT-CALD1 3′UTR but not in cells transfected with MUT-MIR100HG and MUT-CALD1 3′UTR (Figures 8I,J). Thus, miR-142-5p directly targets the sequences of MIR100HG and CALD1. Subsequently, we analyzed the possible role of miR-142-5p in mediating the regulation of bladder cancer cell function by MIR100HG. qPCR results showed that the miR-142-5p mimic effectively induced the expression of miR-142-5p in 5,637 cells (Figure 9A). Then, CCK-8 experiment results showed that the miR-142-5p mimic reversed the effect of pCDH-MIR100HG on the proliferation of 5,637 cells (Figure 9B). RT-qPCR results showed MIR100HG overexpression would cause down-regulation of miR-142-5p (Figure 9C). Based on these assays, MIR100HG can bind with miR-142-5p and negatively regulates miR-142-5p expression. In addition, miR-142-5p overexpression significantly reduced CALD1 expression in 5,637 cells (Figure 9E). Moreover, RT-qPCR and western blot results showed CALD1 expression was significantly increased by pCDH-MIR100HG and reversed by the miR-142-5p mimic (Figures 9F,G). These results further revealed the direct targeting relationship among miR-142-5p, MIR100HG and CALD1, and suggested that the MIR100HG/miR-142-5p/CALD1 is involved in the progression of bladder cancer.

## Discussion

Previous studies have mainly focused on the roles of functional genes in disease mechanisms, especially non-coding RNAs, such as lncRNAs and miRNAs. Epigenetic regulation mediated by functional genes in tumor cells is also an important tumorigenic mechanism, which is being explored by increasing number of researchers (Anastasiadou et al., 2018; Slack and Chinnaiyan, 2019; Goodall and Wickramasinghe, 2021). In bladder cancer, a large number of abnormally expressed lncRNAs and miRNAs have been identified, but the mechanism by which they mediate the process of epigenetic regulation is not clear. Therefore, this study used high-throughput technology to perform transcriptome sequencing of bladder cancer samples and explore the changes in the expression profile of lncRNAs in bladder cancer and the role of key lncRNAs in the progression of bladder cancer. Among them, the lncRNA MIR100HG was significantly up-regulated in bladder cancer tissues compared with adjacent tissues, and its high expression was positively correlated with the histological grade and clinical stage of bladder cancer. Moreover, interference with its expression (overexpression and knockout) altered the proliferation, migration and invasion of bladder cancer cells. In addition, we further found that MIR100HG regulated CALD1 expression by targeting miR-142-5p, and the miR-142-5p mimic reversed the regulatory effect of MIR100HG overexpression on the proliferation of bladder cancer cells. These research data provide a new molecular mechanism underlying the occurrence and development of bladder cancer that involves ceRNA regulation.

In recent years, researchers have found that an increasing number of ceRNA networks are involved in the occurrence of diseases, especially tumors. For example, Wang et al. found that the lncRNA UCA1 promotes the malignant phenotype of renal cancer cells by regulating the miR-182-5p/DLL4 axis as a ceRNA (Wang et al., 2020). According to Xiong et al., the H19/let-7/Lin28 ceRNA network mediates autophagy to inhibit the breast cancer epithelial-mesenchymal transition (Xiong et al., 2020). Some researchers have also reported that the ceRNA network based on lncRNAs-miRNAs-mRNAs also predicts the prognosis of cancer patients. For example, Liu et al. identified a 3-lncRNA prognostic biomarker for colorectal cancer by analyzing the ceRNA network (Liu et al., 2020). Li et al. established a 3-lncRNA prediction model for the prognosis of esophageal cancer based on the ceRNA network and Cox regression model (Li et al., 2020c). Therefore, the study of RNA interactions at the molecular level will provide a better understanding of the gene regulatory networks involved in cancer. In the present study, we used the limma package to determine the differentially expressed lncRNAs and mRNAs between bladder cancer tissues and adjacent tissues. Then, using a KM curve analysis, univariate and multivariate Cox analysis, risk model construction, lncRNA-mRNA network, WGCNA co-expression network and PPI network screening, 13 common mRNAs and 3 independent prognostic lncRNAs (ADAMTS9-AS1, ADAMTS9-AS2 and MIR100HG) were identified. The 13 common mRNAs were mainly involved in muscle contraction, muscle cell development, angiogenesis, protein binding, cell matrix connection, vascular smooth muscle contraction pathway, calcium signaling pathway and MAPK signaling pathway. Then 13 common mRNAs and 3 lncRNAs targeting miRNAs were predicted by starBase, and 21 common miRNAs were obtained. Finally, 13 mRNAs, 3 lncRNAs and 21 miRNAs were used to construct a ceRNA network, and a key ceRNA network MIR100HG/miR-142-5p/CALD1 was selected. Based on this information, MIR100HG/miR-142-5p may affect the biological processes and staging of bladder cancer by regulating CALD1 expression.

Notably, lncRNAs have been identified as an important pathogenic mechanism for the occurrence and development of various cancers, and bladder cancer is no exception. Therefore, lncRNAs have universal significance in the development of bladder cancer. For example, Dai et al. reported that Lnc-MUC20-9 binds to ROCK1 and functions as a tumor suppressor in bladder cancer (Dai et al., 2020). They also found that Lnc-STYK1-2 regulates the proliferation, migration and invasion of bladder cancer cells by targeting the expression of miR-146b-5p and the AKT/STAT3/NF-kB signaling pathway (Dai et al., 2021). MIR100HG is a multicistronic miRNA host gene, encoding miR-100, let-7a-2 and miR-125b-1 in its third intron (Li et al., 2019a). MIR100HG has been reported to be upregulated or downregulated in different human tumor tissues. MIR100HG is expressed at high levels in triple-negative breast cancer (Chen et al., 2020b), laryngeal squamous cell carcinoma (Huang et al., 2019), gastric cancer (Li et al., 2019b) and colorectal cancer (Li et al., 2019a). In non-small cell lung cancer, its expression is down-regulated compared with that in normal tissues. At the same time, the abnormal expression of MIR100HG in these tumors directly regulates the proliferation, migration and invasion of tumor cells (Yu et al., 2015). However, the role of MIR100HG in the development of bladder cancer is poorly understood. As shown in the present study, MIR100HG is an essential cancer-promoting factor in bladder cancer. Overexpression of MIR100HG increased the proliferation, migration and invasion of bladder cancer. These results indicate that the potential application of MIR100HG in the prediction, diagnosis and treatment of bladder cancer is worthy of further study.

LncRNA interference with gene expression is generally achieved by regulating miRNAs or mRNAs. We established the lncRNA-miRNA-mRNA ceRNA interaction network based on the differentially expressed key mRNAs in the lncRNA-mRNA network and MIR100HG to understand the molecular mechanisms of miRNAs downstream of MIR100HG and found that miR-142-5p may be the ceRNA of MIR100HG. Previous studies have shown that miR-142-5p is a key miRNA involved in the progression of many diseases, such as cancer, osteoarthritis and diabetic retinopathy (Shrestha et al., 2017). In cancer, miR-142-5p functions as an oncogene and tumor suppressor. Overexpression of miR-142-5p in renal cell carcinoma promotes cell proliferation and migration (Zhu et al., 2020). Similarly, overexpression of miR-142-5p in colorectal cancer also promotes cell proliferation and migration (Islam et al., 2018). However, in non-small cell lung cancer and liver cancer, overexpression of miR-142-5p inhibits cell proliferation, migration and invasion *in vitro* and *in vivo*, and induces cell apoptosis (Lou et al., 2017; Wang et al., 2017). At present, few studies have examined the function of miR-142-5p in bladder cancer cells. This study confirmed the direct interaction between MIR100HG and miR-142-5p through dual luciferase assays, and further confirmed that the expression of miR-146b-5p in bladder cancer cells increased after MIR100HG knockout. At the same time, the miR-142-5p mimic significantly reversed the effect of MIR100HG overexpression on the proliferation of bladder cancer cells, which indicates the pathogenic effect of the MIR100HG/miR-142-5p interaction on the development and progression of bladder cancer. In addition, by performing a dual luciferase activity, we also confirmed the direct targeting relationship between miR-142-5p and CALD1, and qPCR experiments confirmed that overexpression of miR-142-5p and si-MIR100HG significantly reduce CALD1 expression in 5,637 cells. At the same time, the miR-142-5p inhibitor reversed the effect of si-MIR100HG on CALD1 expression. Based on these results, MIR100HG regulates the expression of the CALD1 by targeting miR-142-5p. Caldesmon (CALD1) is a cytoskeletal protein that regulates cell morphology and movement through interactions with actin filaments, and is closely related to tumor cell migration, invasion, metastasis and blood vessel formation (Cheng et al., 2021; Yao et al., 2021). Therefore, it has attracted increasing attention of cancer researcher in recent years. CALD1 is up-regulated in different types of tumors. For example, the intracranial metastasis of non-small cell lung cancer cell is strongly correlated with the up-regulation of CALD1(Zhang et al., 2014). The angiogenesis of follicular astrocytoma is closely related to a high expression level of CALD1(Zheng et al., 2004). In bladder cancer, CALD1 is a prognostic biomarker and is associated with immune infiltration (Du et al., 2021). However, our results indicate that CALD1 may be involved in the progression of bladder cancer. Overall, the results from this study indicate that CALD1 is a key gene downstream of the MIR100HG/miR-142-5p axis involved in the initiation and progression of bladder cancer. MIR100HG/miR-142-5p/CALD1 is an important ceRNA network involved in the occurrence and development of bladder cancer.

Inevitably, our research also has some limitations that must be addressed. We performed transcriptome sequencing on bladder cancer tissues and only collected 3 pairs of clinical samples, which strictly requires sequencing data support. Second, although we combined data from TCGA database, a series of bioinformatics methods, and *in vitro* cell experiments to verify our data, this study lacks additional verification using other bladder cancer cells and *in vivo* experiments. Finally, this study did not analyze and elaborate on the signaling pathways regulated by the MIR100HG/miR-142-5p/CALD1 axis in detail. Despite these shortcomings, our research results still show that MIR100HG is an important regulator of the progression of bladder cancer and an independent prognostic factor for bladder cancer to a certain extent, and this axis has become a potential biomarker for the diagnosis and prognosis of bladder cancer. At the same time, the MIR100HG/miR-142-5p/CALD1 axis plays an important role in the progression of bladder cancer.

In summary, through transcriptome sequencing, bioinformatics analysis and analyses of cellular functions, we found that MIR100HG is a new bladder cancer promoter that regulates the expression of the CALD1 gene by targeting miR-142-5p and promotes the proliferation, migration and invasion of bladder cancer cells. These findings provide a new perspective for the study of the pathogenesis and development of bladder cancer and reveal the potential of this network to serve as a new target for the diagnosis, prognosis and targeted therapy of bladder cancer.

## Data Availability

The datasets presented in this study can be found in online repositories. The names of the repository/repositories and accession number(s) can be found in the article/Supplementary Material.
