# Supplementary material for: MIR100HG Regulates CALD1 Gene Expression by Targeting miR-142-5p to Affect the Progression of Bladder Cancer Cells in vitro, as Revealed by Transcriptome Sequencing
Source: Front Mol Biosci. 2022 Jan 21;8:793493. doi: 10.3389/fmolb.2021.793493 (PMC8814626; doi:10.3389/fmolb.2021.793493)
Supplement: Supplementary file 1 [file Table1.docx]

| **lncRNA** | **log2 FC** | **Overall survival** | |
| --- | --- | --- | --- |
|  |  | **Hazard ratio** | **p-value** |
| AC008011.2 | -10.15581342 | 1.35 | 0.045 |
| AL356489.2 | -5.980623213 | 1.40 | 0.025 |
| AP001107.5 | -5.792843381 | 1.49 | 0.0091 |
| ADAMTS9-AS1 | -5.233645148 | 2.20 | 7.3E-07 |
| AC110491.1 | -5.18245111 | 1.47 | 0.011 |
| LINC01013 | -5.033515025 | 1.40 | 0.037 |
| CARMN | -4.83679049 | 1.47 | 0.011 |
| ADAMTS9-AS2 | -4.650034889 | 1.50 | 0.0081 |
| AC025259.3 | -4.474416733 | 1.34 | 0.049 |
| AL513217.1 | -4.216094443 | 0.70 | 0.019 |
| ROR1-AS1 | -4.112758822 | 1.40 | 0.04 |
| MIR100HG | 3.869715715 | 1.40 | 0.045 |
| AP000892.3 | -2.909527328 | 1.50 | 0.0077 |
| AC097347.1 | 4.317134901 | 0.69 | 0.014 |
| AL161630.1 | 11.09112151 | 0.63 | 0.0027 |
| LINC00967 | 14.44679322 | 0.63 | 0.0024 |

Supplementary table 1. Prognosis values of differentially expressed lncRNAs in bladder cancer.
